# Mixed Response to Immunotherapy in Patients with Metastatic Melanoma

**DOI:** 10.1245/s10434-020-08657-6

**Published:** 2020-05-29

**Authors:** Daan Jan Willem Rauwerdink, George Molina, Dennie Tompers Frederick, Tanya Sharova, Jos van der Hage, Sonia Cohen, Genevieve Marie Boland

**Affiliations:** 1 Division of Surgical Oncology, Massachusetts General Hospital, Harvard Medical School, Boston, MA USA; 2 Department of Surgery, Leiden University Medical Center, Leiden University, Leiden, RC The Netherlands; 3 Division of Surgical Oncology, Department of Surgery, Massachusetts General Hospital, Yawkey Center for Outpatient Care, Boston, MA USA

## Abstract

**Background:**

Immunotherapy has improved overall survival in metastatic melanoma. Response to therapy can be difficult to evaluate as the traditionally used RECIST 1.1 criteria do not capture heterogeneous responses. Here we describe the clinical characterization of melanoma patients with a clinically defined mixed response to immunotherapy.

**Methods:**

This was a single institution, retrospective analysis of stage IV melanoma patients who received first-line anti-CTLA-4, anti-PD1, or combination anti-CTLA-4/anti-PD1. Therapy response was assessed via clinical definitions, which consisted of cross-sectional imaging combined with clinical exam. Course of disease, clinicopathological characteristics, and management in patients with a mixed clinical response were analyzed.

**Results:**

In 292 patients (anti-CTLA4 = 63; anti-PD1 = 148, anti-CTLA4/anti-PD1 = 81), 103 were responders (35%), 64 mixed responders (22%), and 125 patients had progressive disease (43%). Of patients with a mixed response, 56% eventually had response to therapy (mixed response followed by response, MR–R), while 31% progressed on therapy (MR–NR). MR–NR patients had higher median LDH (*p* < 0.01), 3 or more organ sites with metastases (*p* < 0.01), and more frequently had M1d disease (*p* < 0.01). Mixed responders who underwent surgery (*n* = 20) had a significantly longer mean OS compared to patients who did not undergo surgery (6.9 years, 95% CI 6.2–7.6 vs. 6.0 years, 95% CI 4.6–7.3, *p* = 0.02).

**Discussion:**

Mixed response to immunotherapy in metastatic melanoma was not uncommon in our cohort (22%). Clinical characteristics associated with progression of disease after initial mixed response included higher LDH, brain metastases, and ≥ 3 organ sites with metastases. Surgical treatment for highly selected patients with a mixed response was associated with improved outcomes.

The advent of immune checkpoint inhibitors has revolutionized the therapeutic landscape of metastatic melanoma and has resulted in significant improvements in patient survival. Anti-cytotoxic T-lymphocyte-associated protein 4 (anti-CTLA-4, ipilimumab) enhances overall survival in metastatic melanoma patients, while anti-programmed cell death protein blocking antibodies (anti-PD-1) have demonstrated improved overall survival.^1^^,^^2^ The combination of anti-PD-1 and anti-CLTA-4 therapy is associated with a higher response rate and a significantly longer survival in patients with metastatic melanoma.^3^ Despite these improvements, the evaluation of individual patient responses to immunotherapy can be complex and unpredictable.^4^^,^^5^ The kinetics and patterns of immunotherapy response are still being fully characterized, but there is a clearly defined subgroup, such as those with stable disease via RECIST (< 20% tumor progression and < 30% tumor regression) who have an intermediate survival.^6^^,^^7^ Nuanced response patterns are poorly detected by current radiographic approaches, such as RECIST, which has led to other immunotherapy-specific radiographic assessments like immunotherapy response RECIST,^8^^,^^9^ which is useful but cumbersome to implement in clinical care. Additionally, real-word clinical descriptions of these nuanced response patterns are still lacking. One current clinical challenge is pseudoprogression, a scenario in which tumors will increase in size but eventually regress.^10^^,^^11^ Additionally, an individual can have simultaneous regression in some tumors with progression in others, termed a mixed response. In other patients, lesions may regress or remain stable for a long period of time (i.e., stable disease), while other patients progress in a single site or organ, termed oligometastatic progression.^12^ These heterogeneous responses are challenging and clinical decisions for these situations are made on a case-by-case basis. Currently, only one study has explored the management of oligometastatic progression in metastatic melanoma, and the literature is comprised of a few individual case reports on patients with a mixed response.^13^^,^^14^ Therefore, we conducted a single center retrospective study on patients with metastatic melanoma treated with first-line anti-CTLA-4 and/or anti-PD-1 therapy who developed a mixed response, defined as simultaneous tumor regression and progression, in order to identify clinicopathological characteristics, define high-risk subgroups, and assess subsequent management and outcomes.

## Methods

### Data Source and Study Design

We conducted a retrospective study of patients with unresectable stage IV melanoma treated at the Massachusetts General Hospital (MGH), spanning September 2011 to November 2019. Informed consent was obtained from all patients in accordance with the Institutional Review Board (IRB).

### Patients

Eligible patients were 18 years of age or older, had histologically confirmed unresectable stage IV cutaneous melanoma according to the eighth edition of the American Joint Committee on Cancer (AJCC) classification [including metastases to skin (M1a), lung (M1b), other visceral sites (M1c), and brain (M1d)], and had an Eastern Cooperative Oncology Group performance-status score of 0 or 1.^15^ Exclusion criteria included previously treated melanoma, ocular melanoma, and missing medical records. Staged patients were treated with first-line immune checkpoint inhibitors (anti-PD-1 or anti-CTLA-4 monotherapy or combined anti-PD-1/anti-CTLA-4 therapy,) according to standard therapeutic doses and cycles. All patients underwent standard of care follow-up at MGH, consisting of radiographic assessment every 12 weeks, clinical evaluation by the involved oncology team, and assessment by the treating medical oncologist in which physical exam and laboratory values were assessed.

### Clinical Variables

Demographic variables (age, gender, race, and ECOG status) were extracted from the electronic medical record (EMR). Primary tumor characteristics were extracted from the dermatopathological report [Breslow thickness (mm), ulceration, location of primary tumor], pathological data were collected on metastatic melanoma lesions [number of sites of metastasis, metastatic mutational status (BRAF V600)]. Lactic acid dehydrogenase (LDH) values were collected from laboratory results. Information on timing of radiation and type/date of surgery was obtained from the EMR.

### Assessment

Treatment response was assessed with computed tomography (CT scan) and, if suitable, magnetic resonance imaging (MRI) by a certified radiologist and included the evaluation of non-lymph node metastatic lesions ≥ 5 mm in the long axis, brain metastases ≥ 2 mm in the long axis, and lymph nodes with ≥ 15 mm in the short axis. Tumor burden was defined as the total sum of all measured lesions. We classified response to treatment into three groups: responders (metastatic lesions regressing and no presence of recurrences or new lesions), mixed responders (simultaneously regressing and progressing metastatic lesions) and non-responders (progressive metastatic lesions without any sites of tumor regression). Mixed response to first-line immunotherapy was measured during the first three follow-up scans. The course of disease in patients with a mixed response was divided into three cohorts: (1) mixed response followed by response (MR–R), (2) stable mixed responder (SMR), and (3) mixed response followed by progression (MR–NR). Subsequent analysis was undertaken for the MR–R and MR–NR groups. The SMR group was excluded due to small cohort size (*n* = 6 patients). An overall survival (OS) analysis was performed on the MR–R and MR–NR groups, defined as the time between metastatic disease confirmation and the date of last follow-up or date of death. An additional survival analysis was performed for the entire mixed response cohort treated with or without subsequent surgery to assess survival outcomes.

To compare our clinical response categories with tumor response measurements according to standard guidelines, we performed treatment response evaluation in a subset of patients with available Response Evaluation Criteria in Solid Tumors (RECIST), version 1.1: complete response (CR, 100% disappearance of target lesion), partial response (PR, ≥ 30% decrease in tumor size), progressive disease (PD, ≥ 20% increase of lesion size), and stable disease (SD, < 30% tumor decrease and < 20% increase in tumor size).

### Statistical Analysis

Descriptive analysis was performed to identify frequencies of demographic variables, clinicopathological variables, and recurrence events for selected patients. Observed frequencies of characteristics were compared between the mixed response groups using a Chi square test, Fisher’s exact test, or Wilcoxon rank test when appropriate. Overall survival curves (OS) were estimated with the Kaplan–Meier method and compared with the log rank test for each response to therapy group. *p* values were two-sided and a *p* value less than 0.05 was considered to be statistically significant. All statistical analyses were conducted using IBM SPSS Statistics version 24 (Armonk, New York) and Stata/IC 13.1 (College Station, TX).

## Results

**Fig. 1:**
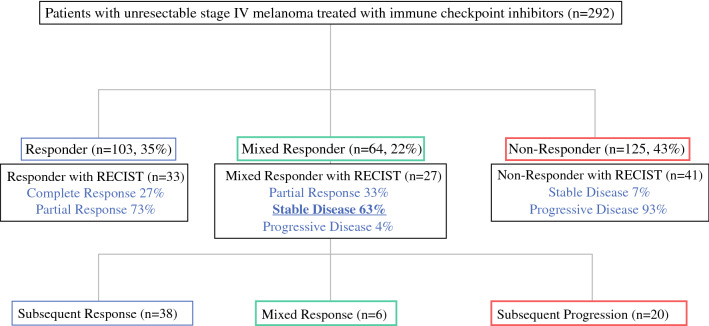
Experimental selection scheme according to clinical classification and RECIST 1.1. Two hundred ninety-two patients with metastatic melanoma were classified by combined clinical/radiographic findings into responder categories: responder (blue), mixed responder (green), and non-responder (red). The black boxes compare RECIST 1.1 categories in a subset of the clinically defined cohorts and demonstrate that the majority of the mixed responders fall into the stable disease (SD) category according to RECIST 1.1. Within the mixed responder cohort, we categorized subsequent response from the time of clinical mixed response

Between 2011 and 2019, a total of 292 patients were diagnosed with unresectable stage IV melanoma and enrolled into our translational protocol. Of these patients, 148 received anti-PD-1 monotherapy (51%), 63 were treated with anti-CTLA-4 monotherapy (22%), and 81 were treated with anti-PD-1/anti-CTLA-4 combination therapy (28%). During treatment, 103 patients were classified as responders (35%), 64 patients demonstrated a mixed response (22%), and 125 patients progressed on therapy (43%, Fig. 1). For patients who had a mixed response, a total of 38 patients subsequently responded to therapy (MR–R), 22 patients eventually progressed on therapy (MR–NR), and 6 patients had a stable mixed responder (SMR). In a subset of patients with RECIST 1.1 data available, a comparison of our clinical categories to RECIST was conducted (*n* = 101 patients). Defined clinical response definitions closely mirrored RECIST findings, as all clinical responders (*n* = 33) correlated to RECIST responders (CR 27%, PR 73%), and similar findings were observed with clinical non-responders (*n* = 41) (SD 7%, PD 93%). Clinical mixed responders (MR–R *n* = 14; MR–SMR *n* = 4; MR–NR *n* = 9) were most commonly categorized into the RECIST 1.1 stable disease group (SD = 63%).

**Fig. 2:**
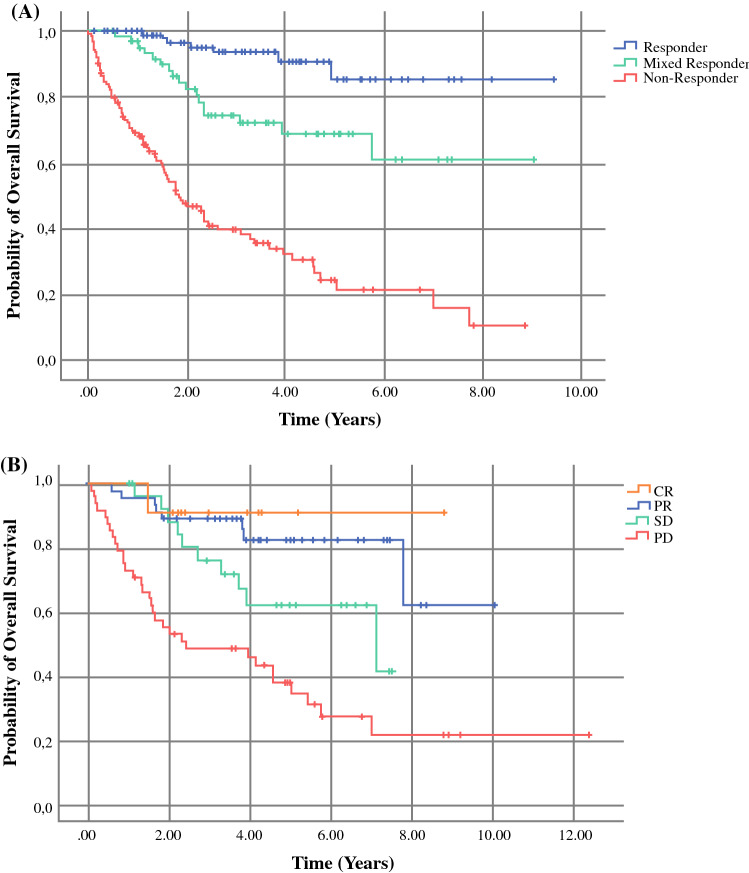
Overall survival. **A** Kaplan–Meier overall survival per clinical response categories, and **B** by RECIST 1.1. **A** Utilizing our clinical categories, the mixed responders (green) have an intermediate response as compared to responders (blue) and non-responders (red). **B** The patients with a partial response (PR) and stable disease (SD) by RECIST 1.1 have an intermediate survival as compared to those with a complete response (CR) or progressive disease (PD)

Unsurprisingly, overall survival analysis demonstrated best outcomes for clinical responders (mean OS of 8.6 years; 95% CI 7.9–9.3) and worst outcomes for clinical non-responders (mean OS 3.2 years; 95% CI 2.5–3.8). There was an intermediate OS for the clinical mixed response group (mean OS 6.6 years; 95% CI 5.6–7.6) (Fig. 2A). Overall survival according to RECIST 1.1 demonstrated the best mean OS of PR in 24 patients (7.5 years; 95% CI 6.8–8.3) median follow-up 3.8 years (IQR 3.1–5.4) and for 9 patients with CR (4.8 years; 95% CI 4.1–5.5) median follow-up 2.7 years (IQR 2.2–4.2). An intermediate mean OS was seen in 20 patients with stable disease (4.5 years; 95%, 3.8–5.7) with median follow-up of 3.5 years (IQR 2.0–4.9), and the worst mean OS was seen in 38 patients with PD (3.5 years; 95% CI 2.5–4.5) and a median follow-up of 3.5 years (2.5–4.5) (Fig. 2B). The observation of an intermediate OS for RECIST 1.1 stable disease and for clinically defined mixed responders supports the previously demonstrated overlap between RECIST 1.1 SD and our mixed responder patients (63% overlap).

**Fig. 3:**
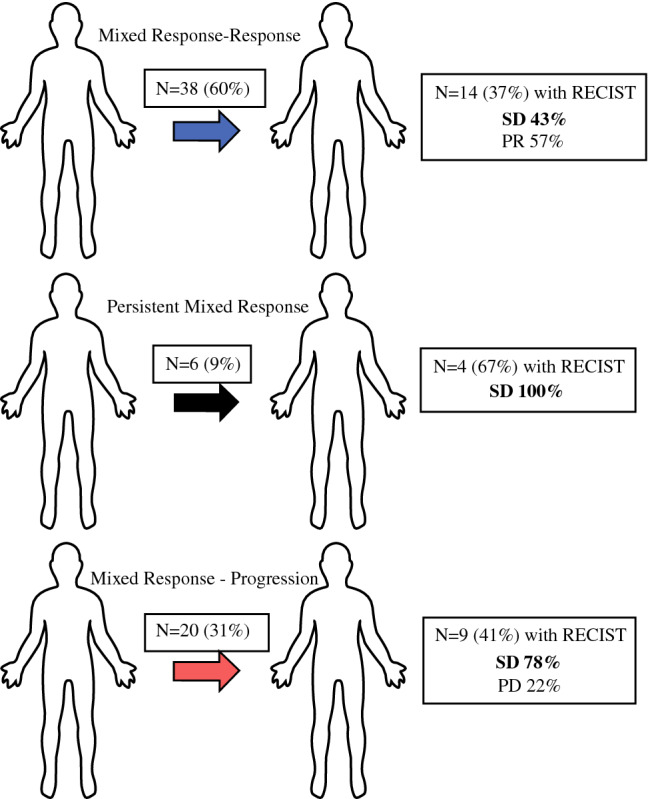
Clinical mixed response group. **A** Within the mixed response clinical group, those with a mixed response and subsequent response (MR–R) comprised 59% of the cohort. A subset of these patients had RECIST 1.1 data for comparison: 43% had SD and 57% had a PR by RECIST. **B** In the subset (9%) of mixed responders with a persistent mixed response, RECIST 1.1 evaluation categorized them as SD (100%). **C** In the mixed responder subset with eventual progression (MR–NR) (31%), RECIST 1.1 data was available in a subset. These patients were characterized as either SD (78%) or PD (22%)

A total of 27 patients who developed a mixed response had RECIST data available at the moment of mixed response confirmation. Of these, 14 patients with MR–R were categorized as SD (43%) versus PR (57%) (Fig. 3). In the sustained mixed response category, 4 (67%) patients had RECIST values for comparison and all (100%) were classified as SD, while 9 patients (41%) in the MR–NR group showed either SD (78%) or PD (22%). Regarding treatments in the mixed response group, patients with an initial mixed response and subsequent progression (MR–NR) were more frequently treated with anti-PD-1 monotherapy (73%) as compared to mixed responders with subsequent response (MR–R, 39%) although this was not statistically significant (*p* = 0.16). On the other hand, MR–R were more often treated with anti-PD-1/anti-CTLA-4 combination therapy (25% vs. 9%), but this was also not significantly different (*p* = 0.06).

**Fig. 4:**
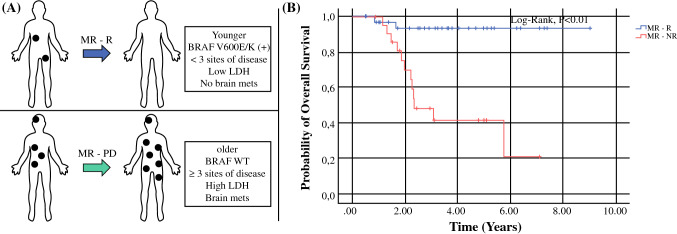
Description of mixed responder categories. **A** Schematic of mixed responders—mixed responders with subsequent responses were more likely to be younger, have BRAF V600E/K mutations, have fewer than 3 sites of disease, low LDH, and no brain metastases. **B** Kaplan–Meier overall survival curves for mixed responders with subsequent response (MR–R) versus those who subsequently progress (MR–NR)

Demographic variables, gender, race, ECOG performance status, location of disease, tumor histology, tumor ulceration, and Breslow thickness, were similar between MR–R versus MR–NR (Table 1). Patients in the MR–NR group as compared to the MR–R group, were older (*p* = 0.03) with a median age of 75 years (IQR 67.5–81.3), had a higher median LDH at mixed response confirmation (*p *< 0.01), more often had brain metastases (i.e., stage M1d; *p *< 0.01), had a higher number of total disease sites (*p *< 0.01), and a trend was seen in BRAF wild-type involvement (*p* = 0.05). In addition, the estimated tumor burden at mixed response was not significantly different between the groups (*p* = 0.11). Furthermore, the median time between mixed response and new response was shorter in MR–NR (141 days IQR 74–265) as compared with MR–R (260 days, IQR 98–434), but was not statistically significant (*p* = 0.09). A schematic of the mixed responder categories is displayed (Fig. 4A). Kaplan–Meier survival curves showed a mean OS of 8.6 years (95% CI 7.9–9.2) for patients with MR–R, 4.0 years (95% CI 2.0–6.0) for patients with a sustained mixed response (not shown), and 3.9 years (95% 2.8–4.9) for MR–NR (*p *< 0.01) (Fig. 4B).

**Table 1:** Clinical characteristics of mixed response group

	MR–R (*n* = 36)	MR–NR (*n* = 22)	*p* value
Median age	67 (29–85)	75 (48–85)	**0.03**
ECOG			
< 1	28 (78)	12 (55)	0.06
≥ 1	8 (22)	10 (45)	
Gender			
Female	10 (28)	4 (18)	0.53
Male	26 (72)	18 (82)	
Location primary			
Trunk	8 (22)	6 (27)	0.55
Lower ex	9 (28)	7 (32)	
Head/neck	10 (28)	3 (14)	
Penis	NA	1 (5)	
Vulva/vaginal	1 (3)	2 (9)	
Unknown	7 (19)	3 (14)	
Tumor histology			
SSM	7 (19)	5 (23)	0.76
Nodular	9 (25)	4 (18)	
Acral	NA	1 (5)	
Mucosal	1 (3)	NA	
Desmoplastic	1 (3)	NA	
Unknown	18 (50)	12 (54)	
Ulceration			
No	12 (33)	8 (36)	0.99
Yes	13 (36)	8 (36)	
Unknown	11 (31)	6 (27)	
Median Breslow (mm)	4.1 (0.4–8.3)	3.2 (0.7–42)	0.64
M stage			
M1a	5 (14)	1 (5)	0.39
M1b	11 (31)	2 (9)	0.1
M1c	12 (33)	6 (27)	0.77
M1d	7 (19)	13 (59)	**< 0.01**
No. sites mets			
No	25 (69)	6 (27)	**< 0.01**
Yes	11 (31)	16 (73)	
Mutational status			
BRAF V600E/K	14 (39)	4 (18)	**0.05**
NRAS	7 (19)	8 (36)	0.41
WT	7 (19)	8 (36)	0.53
Not tested	3 (8)	NA	0.27
Total mutations ≥ 5	7 (19)	3 (14)	0.48
Median LDH	183 (148–213)	212 (190–247)	**< 0.01**

*MR–R* mixed response to response, *MR–NR* mixed response to non-response, *ECOG* Eastern Cooperative Oncology Group, *SSM* sustained mixed response, *BRAF V600E/K* B-Raf proto-oncogene V600E/K mutation, *NRAS* NRAS proto-oncogene, *WT* wild type, *LDH* lactate dehydrogenase

Bold values indicate *p* < 0.05

Patients with a subsequent response tended to be younger (*p* = 0.03), were less likely to have M1d disease (*p* < 0.01), had fewer that 3 sites of metastases (*p* < 0.01), were more likely to have a BRAF V600E/K mutation (*p* = 0.05), and were less likely to have an elevated LDH (*p* < 0.01)

**Fig. 5:**
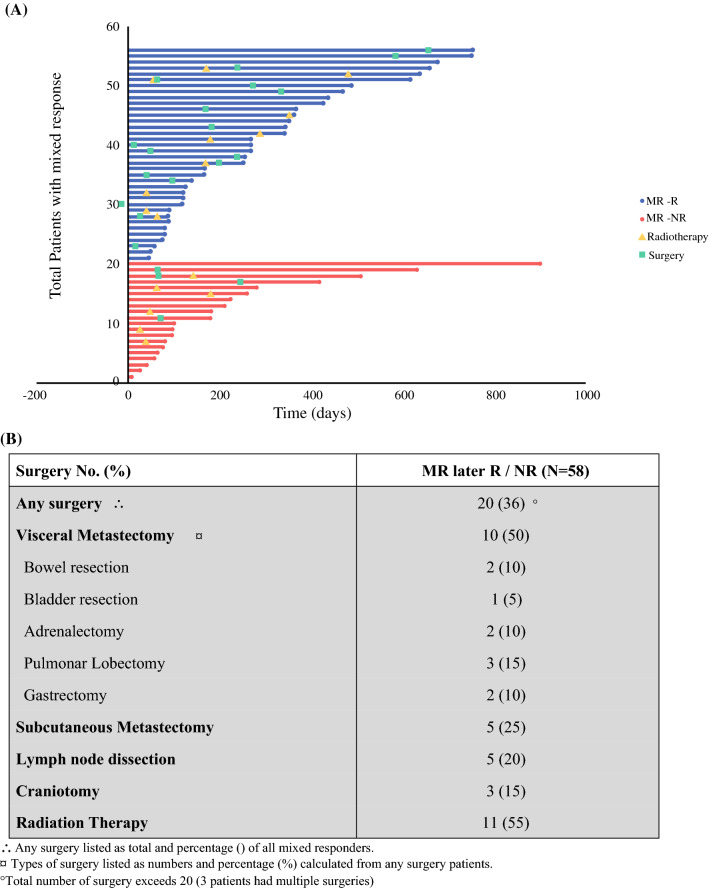
Management of mixed responders. **A** Swimmer plot showing subsequent treatments in mixed responders. MR–R (blue), MR–NR (red), radiation (yellow triangle), surgery (green square). **B** Surgery in patients with a mixed response. In our cohort, 36% of patients had surgery after being categorized as mixed responders. Of these patients, 50% had visceral metastasectomy, 25% had a subcutaneous metastasectomy, 20% had a lymph node dissection, and 15% had a craniotomy

Between the onset of mixed response and a new clinical response, a total of 10 (28%) patients with MR–R received radiotherapy versus 6 (27%) patients with MR–NR (Fig. 5A). Significantly more patients received surgery in the MR–R group (*n* = 16) compared to the MR–NR group (*n* = 4; *p* value < 0.01). The majority of patients with a mixed response who received surgery underwent visceral metastasectomy (50%), while the remainder underwent subcutaneous metastasectomy (25%), lymph node dissection (20%), or craniotomy (15%) (Fig. 5B). A univariate comparison between mixed responders who subsequently had surgery versus mixed responders who did not receive surgical treatment showed similar demographic variables including age, gender, and ECOG status (Table 2). No differences were seen amongst patients who underwent surgery in terms of disease stage, number of organ sites with metastases, or overall tumor burden. However, mixed responders who did not have surgery had a significantly higher median LDH at mixed response (207 U/l IQR 180–237) versus 165 U/l (IQR 141–205) *p* < 0.01) and a significantly shorter time to new response [3.9 months (IQR 2.6–8.9) versus 11.6 months (IQR 6.7–19.3; *p *< 0.01)]. Finally, survival analysis demonstrated a mean OS of 6.0 years (95% CI 4.6–7.3) for mixed responders without surgery and a longer mean OS of 6.9 years (95% CI 6.15–7.6) for mixed responders who subsequently had surgery (log-rank test *p* = 0.02).

**Table 2:** Clinical characteristics of mixed responders who had subsequent surgery

	No surgery between MR–NR *n* = 38	Surgery between MR–NR *n* = 20	*p* value
Median age (range)—years	72 (48–85)	67 (29–86)	0.1
ECOG			
< 1	24	16	
≥ 1	14	4	0.94
Gender			
Female	7 (18)	7 (35)	0.14
Male	31 (82)	13 (65)	
Metastasis status in stage IV			
M1a	3 (8)	3 (15)	0.41
M1b	11 (29)	2 (10)	0.18
M1c	11 (29)	8 (40)	0.77
M1d	13 (34)	7 (35)	1
No. of organ sites w metastasis ≥ 3			
No	19 (50)	12 (60)	0.33
Yes	19 (50)	8 (40)	
Median LDH at MR confirmation	165 (141–205)	207 (180–237)	**< 0.01**
Total tumor burden at MR confirmation	59.5 (36.8–87.8)	58 (24.8–108.1)	0.96
Time between MR and new response (months)	3.9 (2.6–8.9)	11.6 (6.2–19.2)	**< 0.01**

*MR* mixed response (or mixed responder)

Bold values indicate *p* < 0.05

There was no statistical difference between mixed responder groups who subsequently had surgery except a higher median LDH in the MR–NR group (*p* < 0.01) and a shorter time to next response (i.e., progression) in the MR–NR group (*p* < 0.01)

## Discussion

In this retrospective study we analyzed response to immune checkpoint inhibitors in advanced metastatic melanoma patients, and categorized response to therapy into 3 clinical categories: (1) clinical responders, (2) mixed responders, and (3) clinical non-responders. In our cohort, a mixed response to immunotherapy was not uncommon, 22% (*n* = 64), while responses and non-responses were seen in 35% (*n* = 103) and 43% (*n* = 125) of patients, respectively. Direct comparison of our clinical response categories with RECIST 1.1 suggested that our mixed responder cohort aligned most closely with the RECIST stable disease category (63%) and was associated with intermediate survival outcomes.

The mixed responder state was not definitive, as most of the patients with a mixed response eventually developed either a response to therapy (MR–R; 59%) or progression (MR–NR; 31%). Clinical variables associated with MR–NR were a higher median age, higher median LDH at mixed response confirmation, stage M1d, BRAF wild-type tumoral status, and 3 or more organ sites with metastasis. Regarding management in the mixed responder category, patients with a mixed response who went on to respond to therapy (MR–R), were significantly more likely to be treated with surgery as compared to patients in the MR–NR group (*p* < 0.01), likely due to a more favorable phenotype. The types of surgery included visceral metastasectomy (50%), subcutaneous metastasectomy (25%), lymph node dissection (20%), or craniotomy (15%). Unsurprisingly, patients who received surgical treatment had an improved OS as compared to patients who did not undergo surgery (*p* = 0.02). Patients who underwent surgery tended to have a less aggressive disease (i.e., lower LDH, longer period to new disease development). Other studies have shown that surgical treatment in patients with less aggressive heterogeneous responses, such as oligometastatic progression and mixed response to therapy can render patients disease free.^13^^,^^16^^–^18 This potentially supports the added value of our clinical classification system in identifying patients who might benefit from surgical treatment in our mixed response cohort. While surgical decision-making is nuanced, our clinical practice generally supports an observation period for patients with stable disease or mixed response with re-assessment with serial imaging (usually at 3 months). Surgery is favored in patients with no systemic therapy options (i.e., BRAF WT, ongoing immunotherapy toxicity) or those with progressive symptoms. In other settings, surgery is considered on a case by case basis in the context of their overall disease stability/progression.

Clearly the kinetics and heterogeneity of immune checkpoint inhibitor responses are insufficiently captured by RECIST 1.1, which is cumbersome to use in real-world clinical management outside of clinical trials. However, we found that our mixed responder cohort was enriched for RECIST stable disease, with an intermediate survival outcome, and we show that these responses are dynamic and can evolve over time. Interestingly, our work suggests that the mixed responder state is dynamic (ranging from 2.6 to 19.2 months) with the majority of patients transitioning into a definitive response category (R or NR) with associated differences in outcomes. The aim of this current work was to describe the characteristics of the low- versus high-risk groups to assist in risk assessment and clinical decision-making in real-world practice, particularly as it relates to selecting surgical candidates. Our study was limited by the retrospective nature of analysis and small sample size. Despite this, the clinical mixed responder group aligns with the stable disease group according to the RECIST classification, a group in which nuanced clinical decision-making remains a challenge.

## Conclusion

A heterogeneous or mixed tumoral response to immunotherapy in advanced melanoma is not uncommon and represents a dynamic and often transient state, correlating with RECIST 1.1 stable disease. Clinical variables associated with mixed response and subsequent progression of disease were higher median LDH, brain metastases, BRAF wild-type status, and 3 or more organ sites with metastases. In our cohort surgical treatment appeared beneficial for a highly selected group of patients.
